# Supplementary material for: Varidnaviruses in the Human Gut: A Major Expansion of the Order Vinavirales
Source: Viruses. 2022 Aug 23;14(9):1842. doi: 10.3390/v14091842 (PMC9502842; doi:10.3390/v14091842)

## Slide 1
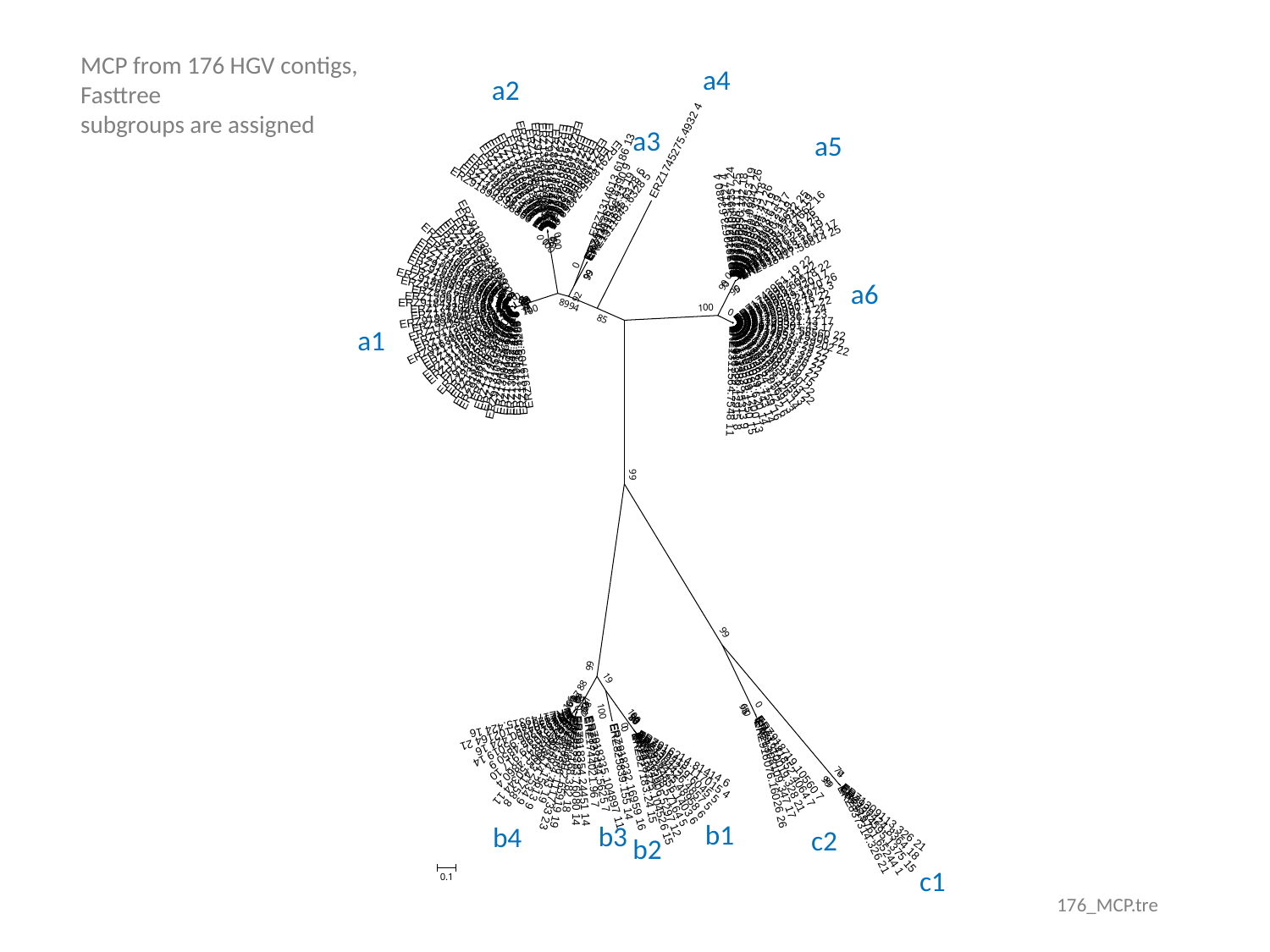

MCP from 176 HGV contigs, Fasttree
subgroups are assigned
a4
a2
a3
a5
a6
a1
b1
b3
b4
c2
b2
c1
176_MCP.tre

## Slide 2
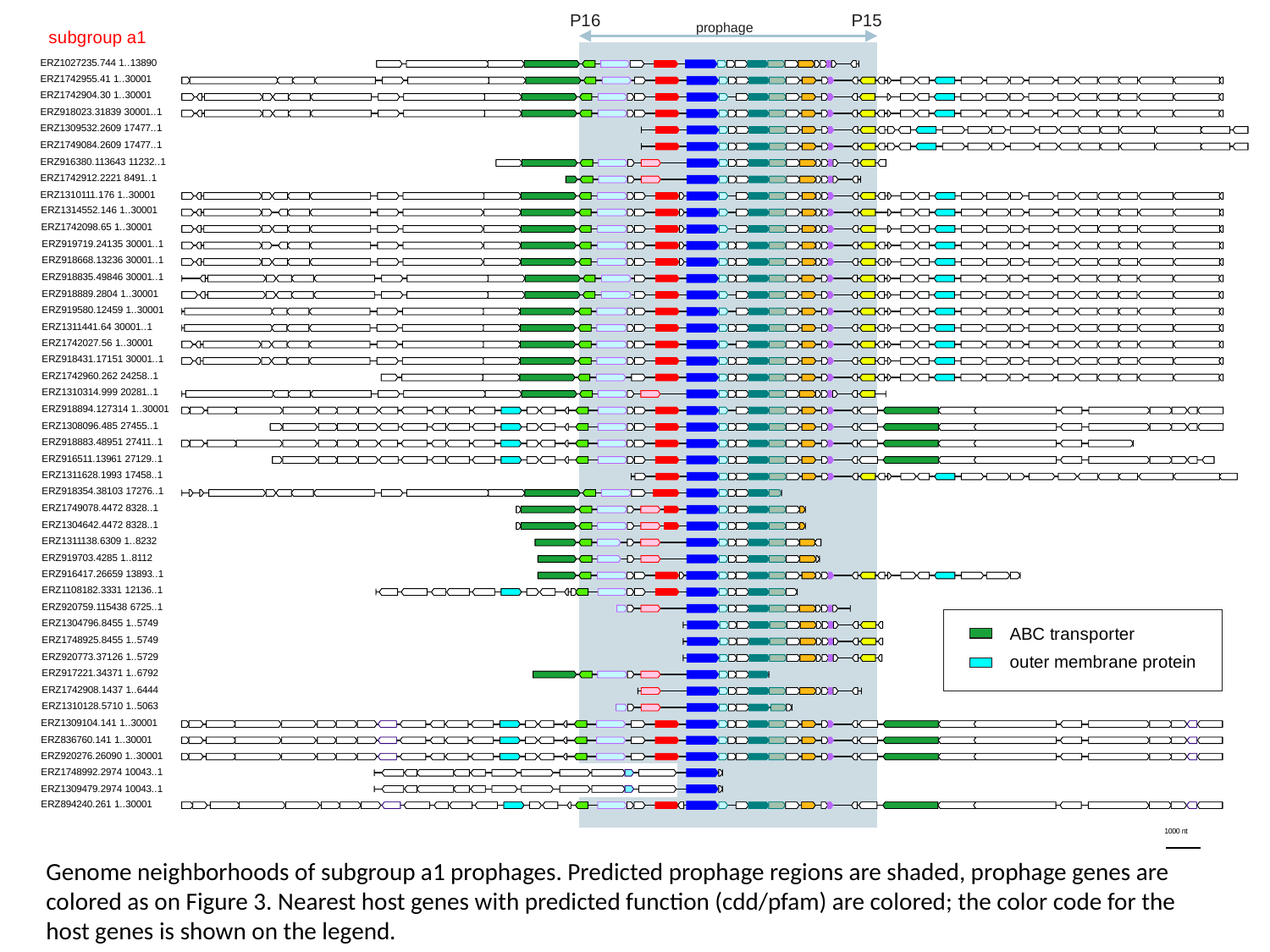

Genome neighborhoods of subgroup a1 prophages. Predicted prophage regions are shaded, prophage genes are colored as on Figure 3. Nearest host genes with predicted function (cdd/pfam) are colored; the color code for the host genes is shown on the legend.

## Slide 3
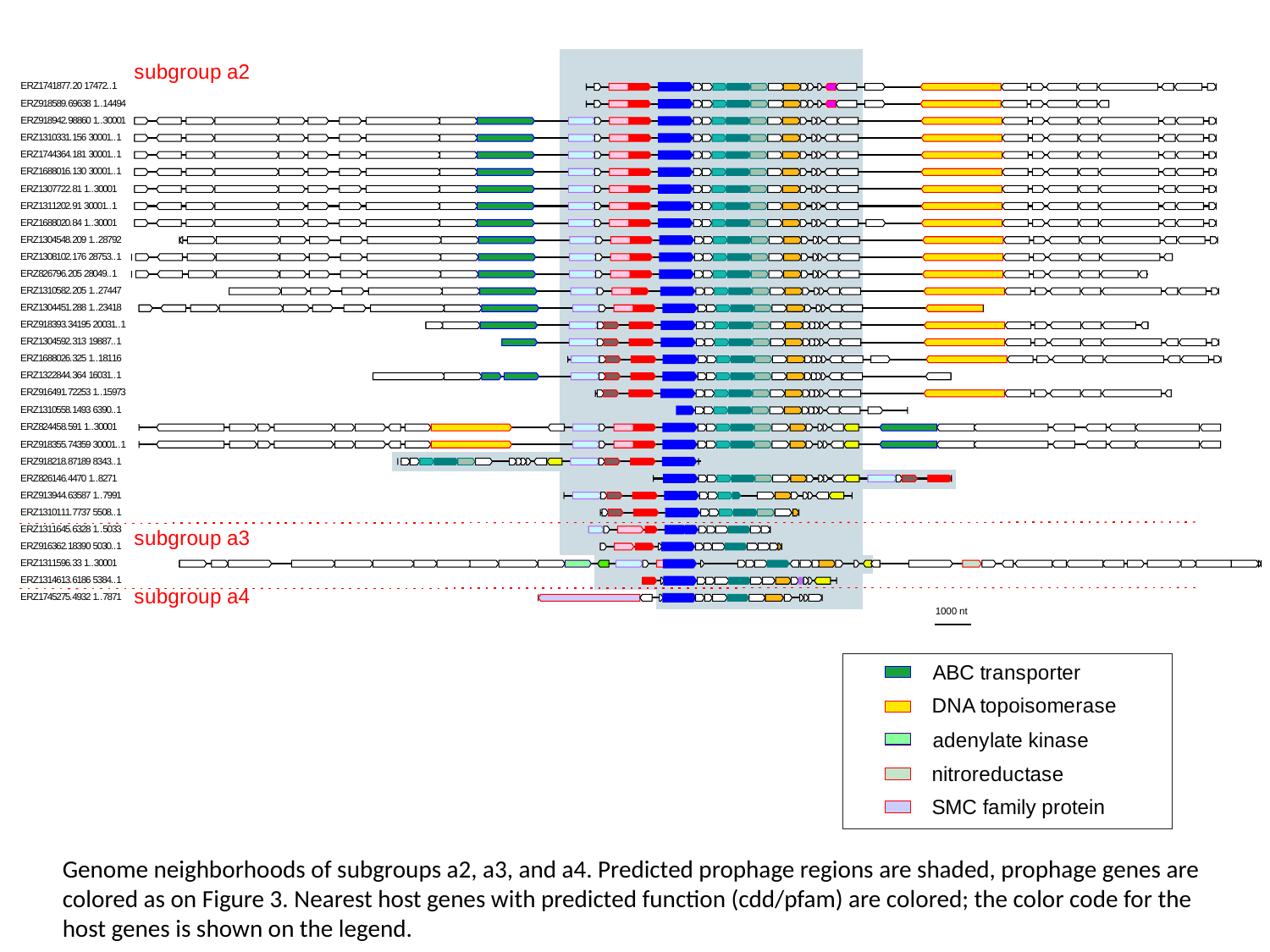

Genome neighborhoods of subgroups a2, a3, and a4. Predicted prophage regions are shaded, prophage genes are colored as on Figure 3. Nearest host genes with predicted function (cdd/pfam) are colored; the color code for the host genes is shown on the legend.

## Slide 4
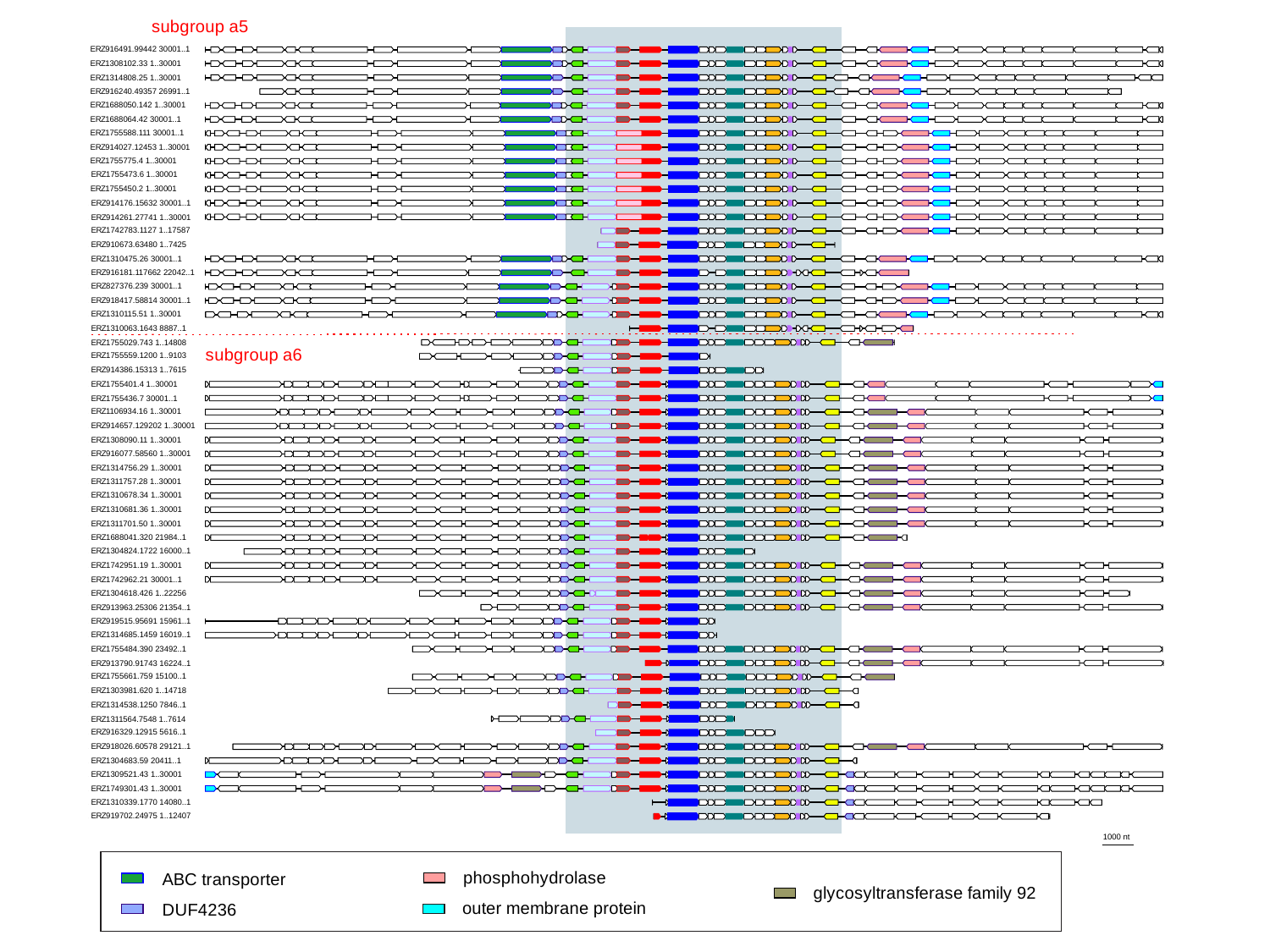

## Slide 5
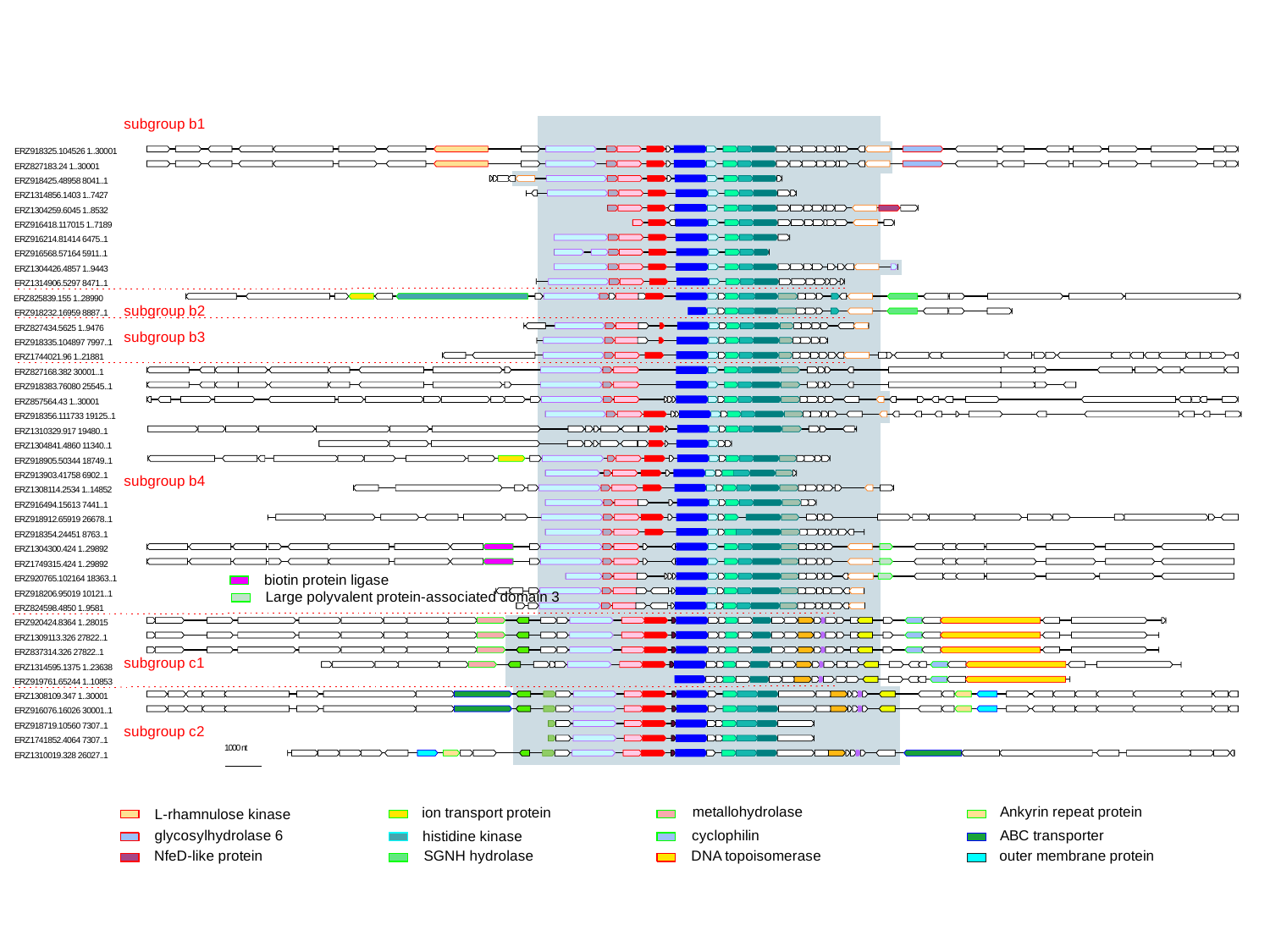

Supplement: Supplementary file 1 [file viruses-14-01842-s001.zip › Supplementary_File_S8.pptx]
